# Is Taurolidine Irrigation Effective in Preventing Surgical Site Infection during Fracture Surgery?

**DOI:** 10.3390/antibiotics13090841

**Published:** 2024-09-04

**Authors:** Shubham Yashwant Dakhode, Woo Sub Kim, Hyun Jin Kim, Seung Yeol Lee

**Affiliations:** 1 Department of Orthopaedics, District General Hospital, Bhandara 441904, India; shubhamdakhode66229@gmail.com; 2 Department of Orthopaedic Surgery, Myongji Hospital, Hanyang University College of Medicine, 55, Hwasu-ro 14beon-gil, Deogyang-gu, Gyeonggi-do, Goyang-si 10475, Republic of Korea; tjqdn19@gmail.com (W.S.K.); weak4329@gmail.com (H.J.K.)

**Keywords:** ankle fractures, infection control, orthopedic procedures, surgical site infection, taurolidine, wound irrigation

## Abstract

Taurolidine, known for its broad-spectrum antimicrobial properties and low toxicity, has shown promise in reducing infections in various surgical settings. However, it has not been extensively evaluated in orthopedic surgery. This study assessed the efficacy of taurolidine irrigation in reducing surgical site infections in patients undergoing ankle fracture surgery. A retrospective review was conducted for patients >20 years old who underwent ankle fracture surgery between March 2016 and March 2023, with follow-ups exceeding 6 months. Patients were classified into the following two groups: those who underwent normal saline (NS) irrigation and those who underwent taurolidine irrigation. Minor infections were defined as requiring additional oral antibiotics postoperatively, while major infections were characterized by hospitalization or reoperation due to infection within 3 months. Of 844 patients, 688 were included. The taurolidine group (*n* = 328) had a significant reduction in minor infections (7.3% vs. 22.5%, odds ratio = 0.410, *p* = 0.028) compared to the NS group (*n* = 360). Major infections were fewer in the NS group (1.2% vs. 0%, *p* = 0.051), but the number of cases was too small for reliable analysis. Taurolidine irrigation significantly reduces the occurrence of minor infections in ankle fracture surgeries when compared to normal saline irrigation.

## 1. Introduction

In orthopedic surgery, infection after fracture fixation poses a serious risk, with rates ranging from 1–2% in closed fractures to 30% in open fractures and peaking as high as 55% [1,2]. Strategies to reduce infection include preoperative screening, aseptic surgical techniques, prophylactic antibiotics, minimizing surgery time, and proper implant selection, along with postoperative measures such as wound care protocols, patient education, and surveillance [3,4].

During surgery, wound irrigation is performed to clear away tissue and dilute bacterial and cellular debris, reducing the risk of surgical site infection [5]. Different solutions, such as normal saline, povidone-iodine, antibiotics, chlorhexidine gluconate, hydrogen peroxide, or taurolidine, are utilized for irrigation in abdominal surgeries to prevent postoperative wound infections [6,7,8,9,10]. In orthopedic surgery, normal saline is the standard irrigation agent due to its non-toxic nature and isotonicity with body fluids. However, alternatives like povidone-iodine and hydrogen peroxide may also be considered [11,12]. In spinal surgeries, vancomycin-, gentamycin-, or streptomycin-based irrigation is performed to prevent infections, while chlorhexidine-based solutions are effective in total joint arthroplasty [13,14,15].

Taurolidine stands out as a highly promising antimicrobial solution due to its efficacy against Gram-positive and Gram-negative bacteria and yeasts/fungi, absence of reported microbial resistance, availability when combined with an anticoagulant, and the rare occurrence of mild side effects [16,17]. Research indicates the effectiveness of taurolidine irrigation in peritoneal lavage based on animal studies [18]. Taurolidine has also been used clinically, but studies on its effectiveness in preventing infections following fracture surgery are lacking.

In musculoskeletal surgery, fractures may present diverse soft tissue conditions that are influenced by location and injury severity, complicating postoperative infection assessment. In focusing on the ankle, this research is significant as it examines the efficacy of taurolidine irrigation, specifically in ankle fracture surgeries, an area with distinct characteristics compared to other orthopedic procedures. The study examined the impact of taurolidine irrigation in ankle fracture patients who underwent similar injury mechanisms and surgical procedures, aiming to assess its efficacy in preventing surgical site infections. The authors hypothesized that taurolidine irrigation would reduce infections following ankle fracture surgery.

## 2. Results

**Figure 1:**
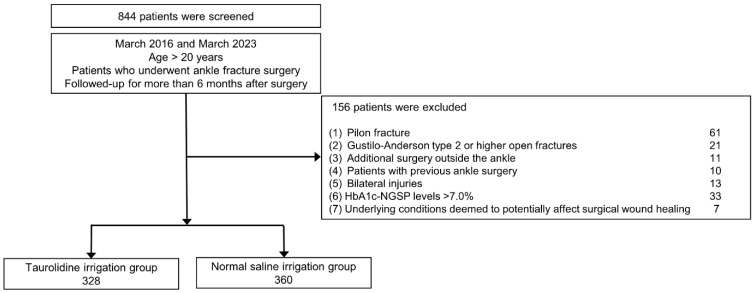
Inclusion and exclusion criteria.

A total of 844 patients met the inclusion criteria and, after applying the exclusion criteria, 688 ankles of 688 patients remained. These patients were classified into the following two groups: the taurolidine irrigation group, which comprised 328 patients, and the NS irrigation group, which included 360 patients (Figure 1). 

### 2.1. Patient Demographics

The mean age at operation was 46.3 ± 16.6 years (range, 20–86 years), and no significant difference in age was observed between the NS and taurolidine irrigation groups (*p* = 0.117). Significantly more diabetic patients were included in the taurolidine irrigation group than the NS irrigation group (*p* = 0.020) (Table 1). 

### 2.2. Infection Outcomes

Among the 688 patients, 46 cases (6.69%) were classified as having minor infections requiring additional antibiotic administration in both groups. In terms of major infections developing within 3 months of surgery, 4 (1.2%) readmissions occurred in the taurolidine irrigation group, with two cases requiring reoperation due to infection. In contrast, no readmissions for major infections within 3 months of surgery occurred in the NS irrigation group (Table 1).

### 2.3. Infection Analysis and Taurolidine Irrigation Impact

Although a statistical analysis was attempted with regard to major infections, the results were deemed unreliable due to the small number of cases. Consequently, further analysis of major infections was not pursued.

In the univariate analysis, without adjusting for other variables, the use of taurolidine irrigation was found to significantly reduce the rate of development of minor infections by 67.8% (odds ratio [OR] = 0.322, *p* = 0.001). Operation time also significantly impacted the need for additional antibiotic administration (*p* = 0.019).

In the multivariate analysis, after adjusting for the effects of sex, age, operative time, and open fractures, the use of taurolidine irrigation independently reduced the need for additional antibiotic administration by 59.0% (OR = 0.410, *p* = 0.028). Other variables did not significantly affect the occurrence of minor infections (Table 2).

## 3. Discussion

This study investigated the effect of taurolidine irrigation on the rate of surgical site infections in ankle fracture surgeries. Based on the results of this study, taurolidine irrigation was shown to significantly reduce the rate of minor infections by 59% compared to normal saline irrigation (*p* = 0.028). This finding supports the hypothesis that taurolidine irrigation can lower the incidence of surgical site infections in ankle fracture surgeries.

Taurolidine, derived from taurine, is a broad-spectrum antibiotic with a unique structure that combines taurinamide and formaldehyde molecules. It effectively targets various bacteria, fungi, and parasites and has antiadhesive and tumor-killing properties, which have been observed in both lab and animal studies [18,19]. Taurolidine can be used locally and is frequently used in abdominal surgeries for its antimicrobial, antiadhesive, and tumoricidal effects. Taurolidine has been shown to inhibit tumor growth within the abdomen when administered intraperitoneally, although it does not have the same effect under the skin [19]. Additionally, both the abdominal and systemic use of taurolidine significantly reduced bacterial spread in an animal model of peritonitis [20]. Taurolidine, in combination with α-tocopherol, decreases oxidative stress in cases of experimental peritonitis [21].

Furthermore, the use of taurolidine intraperitoneally during emergency abdominal surgery significantly reduced septic complications [22]. Taurolidine has also been studied as a lock solution for preventing central venous catheter infections due to long-term use in various medical conditions. Results indicate that taurolidine can effectively penetrate biofilms and act against biofilm cells, in addition to being a strong antibacterial agent [16,17,23]. Side effects have not been reported, making it a popular choice in intensive care and surgical settings [18,19].

Few studies in the field of orthopedic surgery have explored the application of taurolidine in different scenarios. One study identified taurolidine as a promising method for infection prevention when compared to vancomycin. It successfully prevented infections in patients undergoing spinal fusion surgery without any reported side effects, whereas vancomycin showed limited effectiveness, with one infection observed [15]. This study’s results were consistent with those reported here. Another study involving 300 total knee arthroplasty patients subjected to taurolidine irrigation and 300 who did not undergo irrigation showed no significant difference in c-reactive protein (CRP) and erythrocyte sedimentation rate (ESR) levels or infection rates [24]. However, due to the non-specific nature of ESR and CRP, as well as the age demographics of the total knee arthroplasty patients, these results should be interpreted with some caution. 

Surgical site infections (SSIs) following fracture surgeries are common and pose significant risks to patients, ranging from temporary functional impairment to potential limb amputation. Previous research has explored the use of wound irrigation solutions, such as normal saline, povidone-iodine, and chlorhexidine, in the prevention of SSIs, each with varying degrees of effectiveness [5,7,8,10,12]. However, research on the application of taurolidine solution to prevent SSIs, specifically in ankle fracture surgeries, remains lacking. Compared to previous research on spinal surgery (OR 0.83) [15], this study on ankle surgery (OR 0.41) indicated a lower odds ratio for taurolidine irrigation, suggesting its effectiveness in infection prevention. Although differences in study design and patient demographics make study comparisons difficult, the findings, which involved issues with circulation and soft tissue injury, demonstrate the effectiveness of taurolidine irrigation in ankle fracture surgeries.

This study is the first in the field of orthopedics to analyze the impact of taurolidine irrigation on infection prevention following ankle fracture surgery. The findings of this study open new avenues for infection control in orthopedic procedures, where SSIs can have severe consequences on patient outcomes. These findings contribute valuable evidence to the ongoing efforts to enhance surgical site infection prevention strategies by demonstrating a significant reduction in minor infections with the use of taurolidine irrigation.

Despite the promising results, this study has some limitations. First, the retrospective nature of the study may introduce selection bias and limit the ability to establish causality. Second, although patient information was excluded from the datasheet, the sharing of patient IDs may have compromised complete blinding during the data correction and review process. Third, although a statistical analysis was performed for major infections, the results were deemed unreliable due to the small number of cases, and further analysis was not pursued. Additionally, a detailed analysis based on fracture type was not performed. Given the relatively low infection rate, increasing the number of variables might introduce errors in the interpretation of the results. The study also excluded several patient groups, such as those with Gustilo-Anderson type 2 or higher open fractures and uncontrolled diabetes, which might limit the generalizability of these findings. Furthermore, this study did not account for other potential confounding factors, such as variations in surgical technique, postoperative care, and patient compliance, which could have influenced the infection rates, particularly given that two different surgeons performed the surgeries.

Finally, while this study indicates the efficacy of taurolidine in reducing minor infections, the small number of major infections precludes robust conclusions regarding its effectiveness against more severe infections. The limited number of major infections highlights the need for larger prospective studies to confirm these findings and provide more comprehensive evidence on the impact of taurolidine irrigation on both minor and major infections in orthopedic surgeries. Such studies should aim to include a more diverse patient population and consider a wider range of fracture types and surgical techniques to enhance the generalizability and applicability of the results.

## 4. Materials and Methods

This retrospective study was approved by the institutional board of our hospital (IRB no. MJH-2022-12-005). Informed consent was waived due to the retrospective nature of this study.

The medical records of consecutive patients aged >20 years old who underwent ankle fracture surgery with more than 6 months of follow-up between March 2016 and March 2023 were reviewed. Exclusion criteria were as follows: (1) fractures involving the distal tibial metaphysis including pilon fractures, (2) Gustilo-Anderson type 2 or higher open fractures, (3) patients who underwent additional surgery other than the ankle due to multiple fractures, (4) previous ankle surgery, (5) bilateral injuries, (6) uncontrolled diabetes with preoperative hemoglobin A1c levels above 7.0%, and (7) those with underlying conditions deemed to affect surgical wound healing potentially. Age at the time of surgery, sex, hospital stay, tourniquet time, presence of an open fracture, and underlying diseases were obtained from the medical records. Starting from March 2020, taurolidine irrigation was implemented in all patients undergoing surgery. Therefore, patients were classified into the following two groups: (1) the normal saline (NS) irrigation group, up to February 2020, and (2) the taurolidine irrigation group, from March 2020 onward.

Efforts were made to find objective indicators for evaluating the wound infections due to the subjective nature of descriptions in medical records regarding surgical site infections. Orthopedic surgeons with 18, 8, and 3 years of experience conducted a consensus-building session to exclude subjective criteria for determining surgical site infections. For routine surgeries, additional dressing application at the time of stitch removal, typically scheduled around 2 weeks postoperatively, was not performed. However, if oral antibiotics were prescribed after that, it was classified as a minor infection. Meanwhile, a major infection was defined as follows: (1) a history of hospitalization due to infection within 3 months of surgery or (2) undergoing reoperation due to infection within 3 months of surgery.

### 4.1. Surgical Procedures

**Figure 2:**
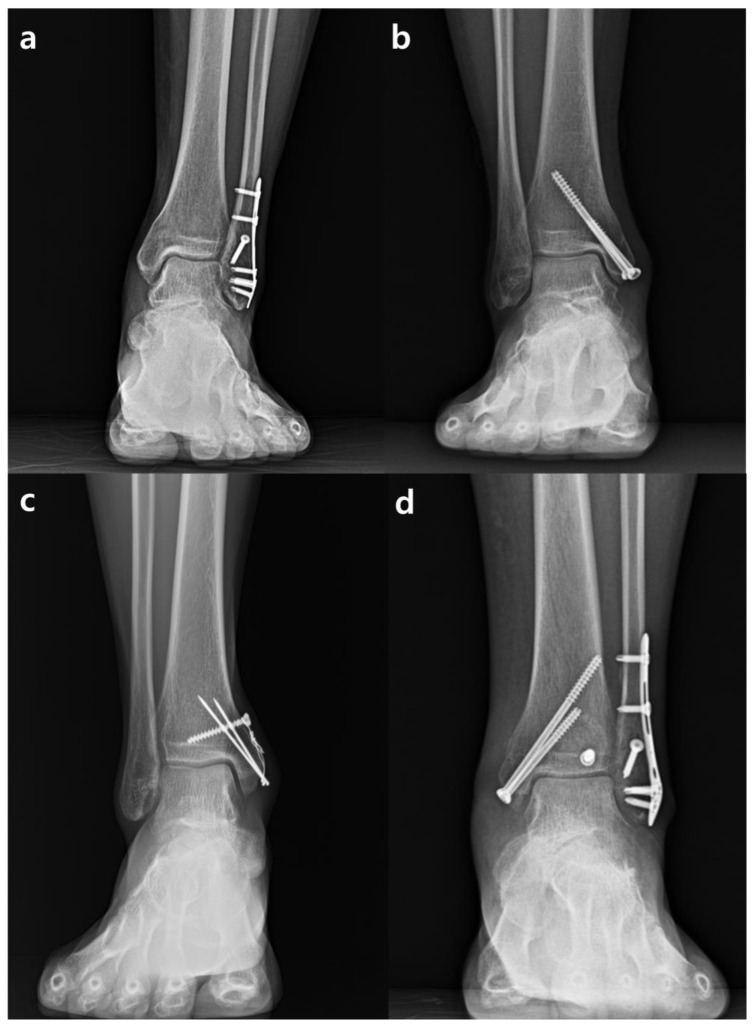
The types and techniques of common ankle fracture surgeries included in this study. Fixation using a lateral locking anatomical plate in a lateral malleolar fracture (**a**). Fixation using two 4.5 mm cannulated screws in medial malleolar fractures (**b**). Fixation using tension band wiring in medial malleolar fractures (**c**). Fixation using arthroscopically assisted cannulated screw fixation in posterior malleolar fracture (**d**).

For ankle fracture surgery, standard surgical techniques were employed. A medial approach was used for medial malleolar fractures, and fixation was achieved with either two 4.5 mm cannulated screws or tension band wiring, depending on the size of the fracture fragment and bone quality. For lateral malleolar fractures, a lateral approach was utilized to insert a locking anatomical plate. In cases where syndesmosis injury was present, a Tightrope^®^ (Arthrex, Naples, FL, USA) was additionally applied. In cases with posterior malleolar fractures, the surgeon performed arthroscopically assisted surgery. Fixation was achieved by inserting a cannulated screw posteriorly through a stab wound created anteriorly (Figure 2).

After fracture fixation was completed and before wound closure, the surgeon performed surgical site irrigation using NS only or NS with taurolidine solution. In the NS irrigation group, irrigation with normal saline at room temperature was performed. In the taurolidine irrigation group, 250 mL of 2% taurolidine solution (Taurolin inj 2%^®^, Samjin Pharm, Seoul, Republic of Korea), maintained at 37 °C, was diluted with 750 mL of normal saline at the same temperature.

### 4.2. Postoperative Antibiotics and Major Infection Management

In South Korea, the use of antibiotics is monitored by health authorities; in fracture surgeries, antibiotic use is restricted to 24 h postoperatively. No empirical antibiotic use was applied in this study. Any additional use of antibiotics beyond this period was considered indicative of a suspected infection.

Regarding the management of infections, patients who underwent surgical treatment for major infections received intravenous antibiotics during their hospital stay. 

### 4.3. Statistical Analysis

R version 4.2.2 (R Foundation for Statistical Computing, Vienna, Austria) and TnF version 4.0 (YooJin BioSoft, Gyeonggi, Republic of Korea) were used for statistical analyses. Data were expressed as medians (interquartile ranges) for continuous variables. For categorical variables, data were expressed as sample numbers and percentages, N (%).

The Wilcoxon rank-sum test was performed to compare continuous variables. The association between taurolidine usage and wound infection after surgery, including major infections analyzed using Fisher’s exact test and minor infections analyzed using the chi-square test, was evaluated. No further analysis was conducted. 

Binary logistic regression analysis was performed to evaluate the effect of taurolidine use on the wound infection rate after surgery, which was identified as additional antibiotic use, readmission within 3 months, and reoperation due to infection. The independent effect of taurolidine use on the development of wound infection was analyzed using multivariable binary logistic regression after controlling for confounding covariates.

## 5. Conclusions

This study shows that the use of taurolidine irrigation during ankle fracture operations effectively reduces minor infections and demonstrates its potential for controlling infections in orthopedic procedures. Despite the clear limitations arising from the retrospective nature of this study, it effectively proves the efficacy of taurolidine in infection control during ankle fracture surgery, which enhances surgical outcomes. Additional prospective studies involving larger and more diverse patient populations are needed to validate further and confirm the effectiveness of taurolidine in musculoskeletal surgeries.

## Figures and Tables

**Table 1:** Patient demographics.

Parameter		Taurolidine Irrigation Group	NS Irrigation Group	*p*-Value
No. subjects (Male/Female)		328	360	
Laterality (Right/Left)		153/175	188/172	0.148
Age at operation (years)		47.4 ± 17.1	45.4 ± 16.2	0.117
Fracture classification				
	Single malleolar	198	198	
	Bimalleolar	38	34	
	Trimalleolar	92	128	
Open fracture		11	4	0.065
OP time (min)		42.5 ± 14.7	41.8 ± 24.9	0.658
DM patients		26	13	0.020
Minor infection		11 (3.4%)	35 (9.7%)	<0.001
Major infection		4 (1.2%)	0 (0%)	0.051

Single malleolar = medial or lateral malleolar fracture; DM = diabetes Mellitus.

**Table 2:** Effects of risk factors on Minor infection.

Variable	Subgroup	N	Case N	Univariable Analysis		Multivariable Analysis	
				OR (95% CI)	*p*-Value	OR (95% CI)	*p*-Value
Taurolidine use		688	46				
	NS irrigation	360	35	Ref.		Ref.	
	Taurolidine irrigation	328	11	0.322 (0.161–0.646)	0.001	0.410 (0.186–0.907)	0.028
Sex		688	46				
	Male	364	28	Ref.			
	Female	324	18	0.706 (0.383–1.302)	0.265	0.741 (0.392–1.401)	0.356
Age		688	46	0.990 (0.974–1.006)	0.215	0.994 (0.977–1.012)	0.531
OP time		688	46	0.979 (0.962–0.997)	0.019	0.990 (0.972–1.008)	0.281
Open fracture		688	46				
	Open	14	1	Ref.		Ref.	
	Closed	674	45	0.930 (0.119–7.271)	0.945	0.564 (0.065–4.857)	0.602

Univariable analysis = univariable binary logistic regression analysis, Multivariable analysis = multivariable binary logistic regression analysis, N = patients in the relevant subgroup, Case N = patients receiving additional antibiotics, OR (95% CI) = odds ratio and 95% confidence interval, NS = normal saline, Ref. = reference subgroup.

## Data Availability

The datasets generated during and analyzed during the current study are not publicly available due to their containing information that could compromise the privacy of research participants but are available from the corresponding author on reasonable request.
